# A Myocardial Segmentation Method Based on Adversarial Learning

**DOI:** 10.1155/2021/6618918

**Published:** 2021-02-26

**Authors:** Tao Wang, Juanli Wang, Jia Zhao, Yanmin Zhang

**Affiliations:** ^1^Department of Pediatric Cardiovascular Medicine, Xi'an Children's Hospital, Xi'an 710003, China; ^2^School of Computer Science and Engineering, Northwestern Polytechnical University, Xi'an 710072, China; ^3^Shaanxi Provincial Institute for Pediatric Diseases, Xi'an Children's Hospital, Xi'an 710003, China

## Abstract

Congenital heart defects (CHD) are structural imperfections of the heart or large blood vessels that are detected around birth and their symptoms vary wildly, with mild case patients having no obvious symptoms and serious cases being potentially life-threatening. Using cardiovascular magnetic resonance imaging (CMRI) technology to create a patient-specific 3D heart model is an important prerequisite for surgical planning in children with CHD. Manually segmenting 3D images using existing tools is time-consuming and laborious, which greatly hinders the routine clinical application of 3D heart models. Therefore, automatic myocardial segmentation algorithms and related computer-aided diagnosis systems have emerged. Currently, the conventional methods for automatic myocardium segmentation are based on deep learning, rather than on the traditional machine learning method. Better results have been achieved, however, difficulties still exist such as CMRI often has, inconsistent signal strength, low contrast, and indistinguishable thin-walled structures near the atrium, valves, and large blood vessels, leading to challenges in automatic myocardium segmentation. Additionally, the labeling of 3D CMR images is time-consuming and laborious, causing problems in obtaining enough accurately labeled data. To solve the above problems, we proposed to apply the idea of adversarial learning to the problem of myocardial segmentation. Through a discriminant model, some additional supervision information is provided as a guide to further improve the performance of the segmentation model. Experiment results on real-world datasets show that our proposed adversarial learning-based method had improved performance compared with the baseline segmentation model and achieved better results on the automatic myocardium segmentation problem.

## 1. Introduction

Congenital heart defect (CHD), also known as congenital heart anomaly or congenital heart disease, is a structural defect of the heart or large blood vessels that occurs at birth. Symptoms vary widely, depending on the specific type of defects [1], ranging from mild to life-threatening. Symptoms typically include shortness of breath, bluish to purple skin color, abnormal weight gain, and fatigue. CHD is usually associated with complications of heart failure without causing chest pain, while most CHD are unrelated to other diseases. CHD is the most common birth defect [2]. In 2015, about 48.9 million people globally suffered from CHD [3]. In different countries and regions, CHD affects 4 to 75 cases per 1,000 live births, and moderate or severe problems can occur in 6 to 19 people per 1,000 [1, 4]. CHD is the main cause of death associated with birth defects. Among many types of CHD, the most common involves the inner walls of the heart, valves, or large blood vessels that pump blood into and out of the heart. Some minor defects do not require treatment, but moderate and severe cases can be effectively treated with catheter-based or cardiac surgery. However, many operations are often required, potentially even including heart transplants. Nevertheless, the death rate from CHD can be greatly reduced, given appropriate treatment is provided.

Cardiovascular magnetic resonance imaging (CMRI) is a noninvasive medical imaging technique used to evaluate the function and structure of the cardiovascular system. By using electrocardiographic (ECG) gated control and high time resolution, regular MRI is adapted to cardiovascular imaging, and its importance is paramount in the evidence-based diagnosis and treatment of cardiovascular diseases [5]. Accurate diagnosis is essential for the development of appropriate treatment regimens for CHD. CMRI can safely provide comprehensive information about CHD, without the use of X-rays or intrusions. This technique is often used in conjunction with other diagnostic techniques, such as echocardiography and diagnostic cardiac catheterization. The use of CMRI for blood pools and myocardium segmentation is a prerequisite for surgical planning and patient-specific heart models for children with complex CHD. The use of existing tools for manual segmentation of 3D images is time-consuming and laborious, which greatly impedes the routine clinical use of 3D heart models. Therefore, automatic myocardial segmentation algorithms and related computer-aided diagnosis systems were developed.

Traditional automatic myocardial segmentation algorithms are generally based on semiautomatic segmentation algorithms. Using prior knowledge, steps such as manual selection of initial contour and initial seed points are automated to realize the automation of the entire cardiac segmentation task. Common myocardial segmentation algorithms include horizontal set segmentation algorithm [6], regional growth segmentation algorithm [7], and threshold segmentation algorithm [8]. However, the segmentation results using this kind of automatic cardiac segmentation algorithms are not ideal, and algorithm robustness is not adequate. With the continuous improvement of hardware equipment and the development of technology, deep learning has been increasingly applied in image processing, resulting in the deep learning-based image segmentation algorithm, surpassing the traditional image segmentation algorithm in many specific tasks [9].

In recent years, with the increase of available data volume and the improvement of computing power, deep learning has made breakthrough progress in various applications in the field of computer vision [10, 11]. Based on these successful experiences, deep learning is now also widely applied in medical image processing [12], including myocardial segmentation. However, common problems in the field of medical image analysis still exist, namely, the low volume of labeled data and networks prone to overfitting. In the field of cardiac segmentation in particular, due to the complex structure of the heart, cardiac labeling is often time-consuming and laborious, which results in the lack of labeled cardiac data. Simultaneously, due to the complex shape of myocardium, myocardium and other surrounding organs and tissues are poorly differentiated in CMR images, and due to the influence of factors such as the tortuous segmentation boundary, room for improvement remains in the final myocardial image segmentation model.

**Figure 1:**
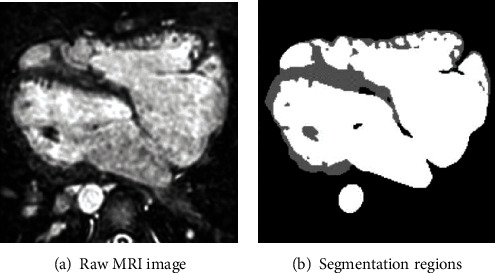
An example of the raw image and its segmentation regions.

This paper applies the idea of antagonistic learning to the segmentation of myocardium, as an attempt to address these issues. Through a discriminant model, additional supervisory information is given to the segmentation model as a guide to further improve its performance. The myocardial segmentation algorithm based on antagonistic learning is mainly composed of two modules: (1) a segmentation network and (2) discrimination networks. Similarly, to the generation of the maximum and minimum game against the network, the segmentation network accepted the input image and generated the segmentation probability graph. The discriminant network received images and corresponding segmentation results simultaneously and determined whether the input segmentation results came from the segmentation network or from manual annotation. We evaluated the method on the HVSMR2016 dataset and the experimental results showed that our method can achieve good results. An example of the raw image and its segmentation regions is showed in Figure 1.

## 2. Related Work

### 2.1. Myocardial Segmentation

Algorithms based on probability models are commonly used to solve the problem of myocardial segmentation among the traditional methods, especially the Gaussian mixture model (GMM) [13, 14]. According to the maximum likelihood (ML) estimation criterion, the expectation maximization (EM) algorithm is usually employed to calculate the parameters in the GMM [15]. On this basis, the Naive Bayes classifier is used to classify each pixel or voxel. Ngo et al. [16] proposed a fully automatic myocardial segmentation method based on depth learning and the level-set algorithm; Mukhopadhyay [17] proposed a fully automatic myocardial segmentation algorithm based on a variational random forest; Tziritas [18] proposed a fully automatic myocardial segmentation algorithm based on the 3D Markov random field; Shahzad et al. [19] proposed a fully automatic myocardial segmentation algorithm that combines the multiple atlas and level-set algorithms. To address the issue of performance and robustness, myocardial segmentation algorithms based on deep learning have been the subject of research. Yu et al. proposed a fully automatic myocardial segmentation algorithm based on 3D fractal convolutional neural networks and dense connection convolutional neural networks [20, 21]. Wolterink et al. [22] proposed a fully automatic myocardial segmentation algorithm based on dilated convolutional neural networks. Avendi et al. [23] applied two deep structures, using convolutional neural networks to automatically detect the left ventricle and a stack automatic encoder to infer the shape of the left myocardium. The inferred shape was then combined into the variability model, to improve the segmentation accuracy. Tran [24] applied fully convolutional network to myocardial MRI segmentation for the first time, extracting ROI regions, and then using the network structure proposed by ROI region pairs to train left and right ventricular segmentation using the stochastic gradient descent (SGD) optimization algorithm. Tao et al. [25] propose a novel shape-transfer GAN for LGE images, which can (1) learn to generate realistic LGE images from bSSFP with the anatomical shape preserved and (2) learn to segment the myocardium of LGE images from these generated images. It is worth to note that no segmentation label of the LGE images is used during this procedure.

### 2.2. Generative Adversarial Networks (GAN)

Generative adversarial networks (GAN), proposed by Goodfellow et al. [26], learn by pitting two neural networks in a zero-sum game with each other. In recent years, GAN have become the most popular learning method of complex probability distribution. They consist of a generator and a discriminator. The goal of the generator is generating samples that are as close to the real data distribution as possible in an attempt to deceive the discriminator, and the goal of the discriminator is to correctly distinguish whether the data belongs to the real distribution or to the generator. The generator and discriminator of conditional generative adversarial networks (CGAN) [27] also use additional condition information, to make the generated data satisfy certain constraints. On the basis of CGAN, Luc et al. [28] used GAN for semantic image segmentation. Xue et al. [29] proposed a novel end-to-end adversarial network architecture called SegAN for MRI image semantic segmentation tasks. Inspired by the original GAN [26], the training process of SegAN is similar to the minimax game, training the segmented network and discriminant network alternately, minimizing and maximizing the objective function, respectively, and combining multiscale loss in SegAN.

## 3. Dataset

The dataset used in this experiment was the HVSMR 2016 dataset. This dataset included 20 MR images with various congenital heart defects, where in 10 cases, the image data and their corresponding manual segmentation labeling have been made public (training set). The remaining 10 cases constitute the test set, which did not include manual segmentation tagging, and the segmentation results needed to be submitted to an online test platform that returned the test results.

The images of this dataset were acquired during clinical practice at Boston Children's Hospital, Boston, MA, USA. Some subjects included in the dataset have undergone interventions. Imaging was done in an axial view on a 1.5 T scanner (Phillips Achieva), without contrast agent, using a steady-state free precession (SSFP) pulse sequence. The subjects breathed freely during the scan, and ECG and respiratory gating were used to eliminate the effects of cardiac and respiratory movements for the duration of the imaging. Manual segmentation of the ventricular myocardium was performed by a trained rater and validated by two clinical experts.

There were three classes of labeling: blood pool, myocardial layer, and background. The blood pool class included the left and right atria, left and right ventricles, aorta, pulmonary veins, pulmonary arteries, and the superior and inferior vena cava. The myocardium class included the thick muscle surrounding the two ventricles and the septum between them. Image dimensions and image spacing vary across subjects, with an average of 390 × 390 × 165 pixels and 0.9 × 0.9 × 0.85 mm, respectively, in the full-volume training dataset.

## 4. Method

### 4.1. Data Preprocessing

The data processing part of the experiment consisted of two steps: data standardization and random block taking.

#### 4.1.1. Data Standardization

The preprocessing process of the experiment was standardized using the *Z*-score:

(1) $$\begin{array}{c} x^{*}=\frac{x-\bar{x}}{s}, \end{array}$$

where *x* is the input image, $\bar{x}$ is the average of the gray value of each voxel in the input image, that is, $\bar{x}=1/n\sum_{i=1}^{n}x_{i}\;$, and *s* is the sample standard deviation of the input image, that is, $s=\sqrt{1/n-1\sum_{i=1}^{n}{\left(x_{i}-\bar{x}\right)}^{2}}$. After standardization, the mean value of *x*^∗^ was 0 and the standard deviation was 1. Data standardization is the most commonly used standardization method and was performed to eliminate the systematic deviation between data as much as possible and be robust to abnormal data values.

#### 4.1.2. Sliding Window Block Taking

Limited by the very small dataset size, data augmentation was a necessary data preprocessing process. In addition, the original 3D image was large in size, therefore, too expensive to input directly into the network for training. Consequently, the method of sliding window block taking was adopted in this experiment. An image block with a size of 64 × 64 × 64 from a standardized input image was extracted along three spatial dimensions with independent uniform distribution, and the corresponding tensor was cut out from the segmentation label according to the corresponding spatial position as the segmentation label of the extracted image block. In order to further expand the size of the training set and to consider the possible direction invariance caused by the acquisition process of MRI, random 90°, 180° and 270° rotations in the axial plane and symmetric flip about the axial plane were also introduced.

### 4.2. Overall Framework

**Figure 2:**
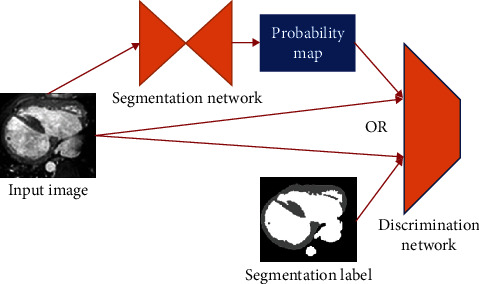
Outline of the cardiac muscle segmentation algorithm based on adversarial learning.

The cardiac muscle segmentation algorithm based on adversarial learning consisted mainly of two modules, namely, the segmentation network and the discrimination network. The segmentation network received the input image and generated the segmentation probability map. The discrimination network then received the image and the corresponding segmentation results simultaneously and determined whether the input segmentation result came from the segmentation network or from manual annotation. The outline of the algorithm is shown in Figure 2. The discrimination network can be regarded as a special loss function, different from the commonly used cross-entropy loss-function and dice loss function, which directly depends on the value of each pixel and defines a complete loss function. The discrimination network analyzed the image and segmentation results jointly and had a deep network structure and a large number of learnable parameters, therefore, it was able to provide advanced guidance information for the segmentation network.

For the input 3D image block and its corresponding segmentation result *x*, *y*, the segmentation probability map *S*(*x*) given by the network was obtained through the segmentation network *S*, using forward reasoning calculation, and the segmentation loss function *J*_seg_ and the adversarial loss function *J*_adv_ were calculated, during training. Similar to the training process of GAN, the segmentation network *S* and the discrimination network *D* were trained in turn, and the parameters of the corresponding network model were updated by the back-propagation algorithm.

In the prediction process, the input 3D image was preprocessed and then several image blocks were extracted in a certain step along the three spatial dimensions and input into the segmentation network *S*, respectively, to obtain the segmentation probability map of the corresponding image blocks. Finally, the segmentation probability maps corresponding to the image blocks at different positions were synthesized, and the segmentation results corresponding to the input 3D images were obtained after postprocessing.

### 4.3. Segmentation Network

A 3D full convolutional neural network was used to segment the cardiac muscle and blood pool. In theory, the full-convolutional neural network can process input images of any size. However, the input image size is directly related to the size of the characteristic tensor of each layer of the network, demanding a lot of runtime memory for oversized input images. Additionally, because the input image and convolution kernel are 3D tensors, the computational complexity will increase significantly with the increase of input image size. Therefore, the sliding window block strategy of size 64 × 64 × 64 was used for the input image, during both training and testing. The training process blocked the input image at random positions. This step can be seen as a form of data augmentation, which expands the size of the training data set and also creates reasonable constraints on the size of the input image block, so that the network model can complete the training process with limited memory and within reasonable calculation time. The input images were taken along three spatial dimensions with overlapping blocks at equal intervals during the test process, and the extracted image blocks were input into the segmentation model to obtain a segmentation probability map; then, the segmentation probability maps at different positions were divided according to the input image block. The spatial position was arranged, and the overlapping part adopted the voting strategy to average the segmentation probability map and finally obtain the segmentation result of the original image.

**Figure 3:**
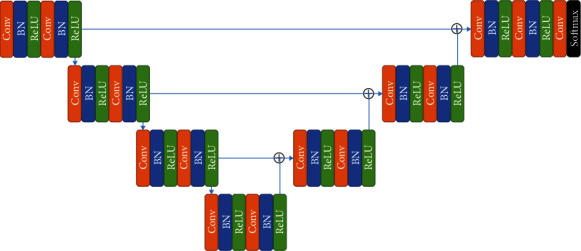
Outline of the segmentation network model.

A full-convolutional neural network model with a structure similar to 3D U-Net was designed in this study. As a segmentation network part, the network structure is shown in Figure 3. The network model used a symmetric encoder-decoder structure to extract the characteristics of the input image and obtain the segmentation probability map through forward reasoning calculation. The network used jump connections, connecting the shallow and deep layers of the network, and was able to simultaneously use high-dimensional semantic features and low-dimensional grayscale, texture, and other image detail features to jointly participate in the final segmentation probability map calculation.

Each scale part of the encoder part was composed of two identical stacked modules, with each module including a convolutional layer with a kernel of 3 × 3 × 3, a step size of 1, a batch normalization (BN) layer, and linear rectification function (Rectified Linear Unit, ReLU). Each time the maximum pooling was performed, the spatial scale of the feature map was halved, but the number of feature channels was doubled to retain a certain amount of information. The decoder part was generally symmetrical with the encoder part and had a similar structure. The kernel size and stride of deconvolution are 2 × 2 × 2 and 2, respectively. The input tensor of each scale consisted of the output of the previous layer after deconvolution, while the output features of the encoder of the corresponding size were spliced together. After deconvolution, the spatial scale of features was doubled, and the number of feature channels was halved. Finally, the network used a convolutional layer with an output channel of 3 and the SoftMax activation function to obtain a segmentation probability map. The three channels represent the three category labels of cardiac muscle, blood pool, and background, respectively.

### 4.4. Discriminant Network

**Figure 4:**
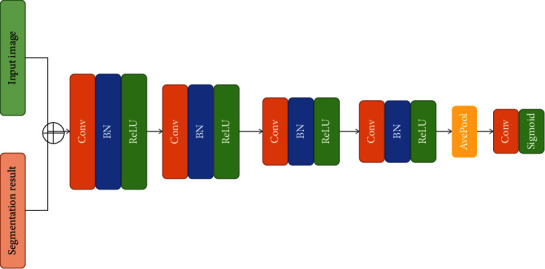
Outline of the discrimination network model.

A full-convolutional neural network model is presented in this paper. The number of layers is shallow, and the structure is similar to the VGG network. The network structure is shown in Figure 4. The basic module consisted of a convolutional layer with a step size of two, followed by batch normalization and a linear rectification activation function. The convolutional layer with a step size of two can extract features and reduce the scale of the feature map. The input of the discriminant network was the input image and segmentation results. After the processing of four basic network modules, the discriminate results were obtained by global average pooling and sigmoid activation function. The output value, ranging from 0 to 1, represented the probability that the segmentation result was derived from the manual annotation.

Compared with the segmented network, the discriminant network was shallower, and the number of parameters used is lower. The reason for this design was that the segmentation network was tasked with the relatively complex task of generating segmentation results, which was the main part of the model. Conversely, the sole output of the discriminant network was one probability value, and too many parameters are easy to over fit, which is not conducive to the convergence of the model.

### 4.5. Loss Function

The most commonly used loss function in image segmentation tasks is the pixel by pixel loss entropy error function. The value of the loss function on each pixel (voxel) was calculated independently. The pixel classification prediction was compared with the standard vector encoded by one-hot to measure the difference between them. The calculation formula of the cross-entropy loss function is shown in equation (2):

(2) $$\begin{array}{c} J_{\text{CE}}=-\frac{1}{N}\underset{\text{classes}}{\sum }\overset{N}{\underset{i=1}{\sum }}g_{i}\ln p_{i}, \end{array}$$

where *N* is the total number of pixels (voxels), classes represent each category, *g*_*i*_ denotes whether the *i*-th pixel is marked as the true label of the current category, and *p*_*i*_ is the prediction probability that the *i*-th pixel is predicted as the current category. It is clear from the formula that the cross-entropy loss function was evaluated separately for each pixel and then the contribution of all pixels was averaged to obtain the final loss value. The segmentation network model combined with cross entropy loss function was the basic method used to address the image segmentation problem by deep learning, and it was also the baseline method of the experiment in this paper.

Adversarial loss function, *J*_adv_ is a minimum-maximization function, defined as equation (3):

(3) $$\begin{array}{c} \min_{S}\max_{D}J_{adv}\left(S,D\right)=E_{x,y\sim P_{data}\left(x,y\right)}\left\{\log D\left(x,y\right)-\log D\left[x,S\left(x\right)\right]\right\}, \end{array}$$

where *S* and *D* are the segmentation network and the discrimination network, respectively, *X* and *Y* are the input image block and the corresponding segmentation result annotation, respectively, *P*_*data*_ is the data distribution composed of the training data set, and *D*(*x*, *y*) is the prediction probability of the segmentation result corresponding to *X*, determined by the segmentation network. When the parameters of the segmentation network were fixed, the discrimination network minimized the binary cross entropy loss function. When the parameters of the discriminant network were fixed, the discrimination network was minimized as follows:

(4) $$\begin{array}{c} -E_{X,Y\sim P_{\text{data}}\left(x,y\right)}\log D\left[x,S\left(x\right)\right]. \end{array}$$

In other words, the segmentation network was induced to produce a more realistic segmentation result.

The cross-entropy loss could effectively measure the difference between the classification prediction value of each pixel and the gold standard, while the counter loss function could comprehensively measure the difference between the predicted image segmentation results and the gold standard from a global perspective, complementing each other. The two loss functions were used at the same time in this study, in order to utilize both their advantages, and the total loss function was defined as shown in equation (4):

(5) $$\begin{array}{c} J=J_{\text{CE}}+\alpha J_{\text{adv}}, \end{array}$$

where *α* is a super parameter used to adjust the relative weight of the above two loss functions. Larger values of *α* lead to larger relative weight of *J*_adv_, which also cause the influence of the adversarial network on segmentation results to be more explicit. On the other hand, smaller *α* values lead to larger relative weight of *J*_CE_, causing the influence of adversarial network on segmentation results to be less explicit. Experimental results showed that when *α* was set as 0.15, the overall performance of the model is optimal. The related parameters are discussed in detail in the discussion part.

### 4.6. Evaluation Index and Implementation Details

The commonly used image segmentation task evaluation index Dice coefficient (DSC) was used to evaluate the performance of myocardial and blood pool segmentation. The definition of DSC is as follows:

(6) $$\begin{array}{c} \text{DSC}=\frac{2\left|X⋂Y\right|}{\left|X\right|+\;|\;Y\;|\;}, \end{array}$$

where *X* and *Y* are the predicted segmentation result and the manually annotated segmentation result, respectively. DSC is a dimensionless number between 0 and 1 that measures the similarity of two sets. High DSC values are associated with close match between the predicted segmentation result of the model and that of manual annotation, meaning better model performance.

The experiment was based on the deep learning framework Keras. The Adam adaptive optimization algorithm was used to complete the training and testing of the network model, using an NVIDIA 1080Ti GPU hardware platform. The network was trained on the HVSMR 2016 dataset. The Leave-One-Out scheme was used in the study, since there were only 10 samples in the training set. One sample was selected as the validation set, and the remaining nine samples were selected as the training set that the 10-fold cross-validation experiment was performed in turn to verify the effectiveness of the proposed method and the discussion experiment of super parameter. The final model was then tested online with the complete training set and the optimized hyperparameter training. The learning rate is 0.001, and the batch size is 16.

## 5. Experiments and Results

We demonstrated the myocardial segmentation algorithm based on adversarial learning and analyzed its effectiveness by conducting ablation experiments. The training datasets of the HVSMR 2016 datasets were used to conduct cross-validation using the leave-one-out method and use the average value of the Dice score to evaluate the performance of the model. The experimental results are shown in Table 1.

**Figure 5:**
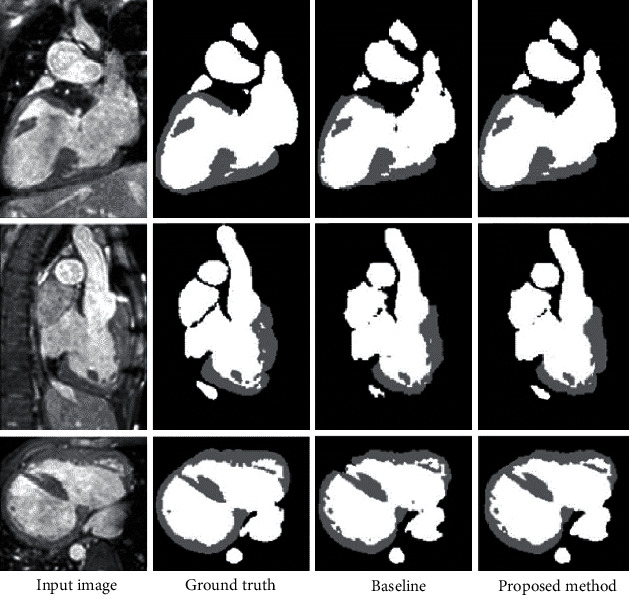
Comparison of segmentation results on the validation set. Each row corresponds to a different case sample. The first column is the CMR image slice of the case sample, the second column is the manually marked segmentation results, and the third column is the baseline model. The fourth column is the segmentation result of the complete model.

The segmentation network shown in Figure 2 was used as the baseline model on the HVSMR 2016 datasets. The discrimination network, as shown in Figure 3, was then added. We conducted relevant experiments again to verify the effectiveness of the discriminative network. Comparing the experimental results in Table 1, it is reasonable to conclude that after using the discriminative network and introducing the adversarial learning mechanism, the performance of the network model in myocardial segmentation was considerably improved and the Dice score increased from 0.712 to 0.753. The improvement of the blood pool segmentation was very small, because the blood pool has a simple shape and no internal texture and structure. The blood pool was also relatively easy to segment compared to the complex-shaped myocardium with a thin layer structure. The baseline model achieved good results, and improved space was relatively small.  Figure 5 shows the segmentation results of the learning-based segmentation of the partial validation set. It can be seen that the network model that introduced the adversarial learning mechanism gained a better segmentation result than the baseline model, thereby achieving more accurate myocardial segmentation.

The network model trained on the HVSMR 2016 datasets was tested online on the test datasets and compared with other methods published in recent years. These methods are mainly divided into two categories based on traditional machine learning and deep learning, as shown in Table 2.

The traditional machine learning algorithms included a variation random forest algorithm proposed by Mukhopadhyay [17], a 3-D MRF model random field proposed by Tziritas [18], and methods combining multiatlases and level sets proposed by Shahzad et al. [19]. Limited to the characteristics of manual design, the overall performance was slightly worse than the methods based on deep learning. However, in terms of deep learning models, the 3D FractalNet proposed by Yu et al. introduced the idea of recursion, with the network model being a complex fractal structure [20]. Wolterink et al. proposed a convolutional neural network model with two-dimensional holes [22] that combines the ideas of multiple perspectives. 3D UNet has a relatively simple structure and a wide range of applications. Even though a difference in performance was still present between the method of 3D UNet + discrimination network proposed in this paper and the optimal method, compared with the baseline model, the performance was notably improved.

## 6. Discussion

**Figure 6:**
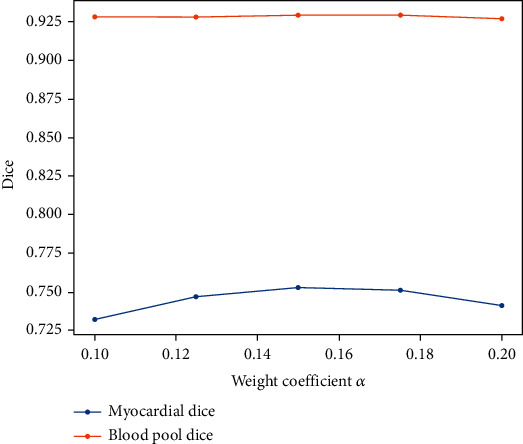
The influence of weight coefficient *α* on model performance for the myocardial layer and blood pool.

The influence of the discriminator on the final segmentation result was affected by using different loss function weight coefficients *α*, further affecting the final average Dice coefficient. In the 10-fold cross-validation experiment conducted on the training datasets, the *α* value was adjusted depending on the average dice coefficient. The performance indexes of different *α* values on the validation datasets are shown in Figure 6. Experiments showed that the model achieves best performance when *α* = 0.15.

## 7. Conclusion

A myocardial segmentation algorithm based on adversarial learning was proposed in this paper, and experiments were designed to comparatively analyze the effectiveness of the adversarial learning mechanism on myocardial tissue segmentation tasks. The introduction of adversarial learning mechanism for model focus on the overall spatial structure and context consistency was successful, and a more accurate segmentation result was obtained. Our method improved the quantitative segmentation performance index considerably, compared with the baseline model.

## Figures and Tables

**Table 1:** Ablation study experimental results of the discriminative network.

Model	Myocardial dice	Blood pool dice
3D UNet (baseline)	0.712	0.926
3D UNet + discrimination network	0.753	0.929

**Table 2:** Quantitative comparison of the method presented in this paper to segmentation performance of different methods from the literature.

Method	Myocardial dice	Blood pool dice
Mukhopadhyay [17]	0.495	0.794
Tziritas [18]	0.612	0.867
Shahzad [19]	0.747	0.885

3D UNet [30]	0.707	0.926
Ours	0.762	0.928
Wolterink et al. [22]	0.802	0.926
Yu et al. [20]	0.786	0.931

## Data Availability

The dataset used in this experiment was the HVSMR 2016 dataset, which is available at: http://segchd.csail.mit.edu/data.html
